# Extreme sampling design in genetic association mapping of quantitative trait loci using balanced and unbalanced case-control samples

**DOI:** 10.1038/s41598-019-51790-w

**Published:** 2019-10-29

**Authors:** Yi Li, Orna Levran, JongJoo Kim, Tiejun Zhang, Xingdong Chen, Chen Suo

**Affiliations:** 1 School of Statistics, Shanxi University of Finance & Economics, Shanxi, China; 2 Laboratory of the Biology of Addictive Diseases, Rockefeller University, New York, US; 3 Department of Biotechnology, Yeungnam University, Gyeongsan, Korea; 4 Department of Epidemiology, School of Public Health, Fudan University, Shanghai, China; 5 Key Laboratory of Public Health Safety (Fudan University), Ministry of Education, Shanghai, China; 6 State Key Laboratory of Genetic Engineering and Collaborative Innovation Center for Genetics and Development, School of Life Sciences, Fudan University, Shanghai, China

**Keywords:** Data processing, Genetic association study, Statistics

## Abstract

It is extremely expensive to conduct large sample size array- or sequencing based genome scale association studies. For a quantitative trait, an extreme case-control study design may improve the power and reduce the cost of variant calling. We investigated the performance of extreme study design when various proportions of samples are selected from the tails of phenotype distribution. Using simulations, we show that when risk genotypes become rare in the population and effect size is relatively small, it is beneficial to carry out an extreme sampling study. In particular, the number of selected cases and controls can even be unbalanced such that power is further increased, compared with a balanced selection. Our application to two data sets: methadone dose data and yearling weight data, demonstrated that similar results for full data analysis can be obtained using extreme sampling with only a fraction of the data. Using power analysis with simulated data and an experimental data application, we conclude that when full data is unavailable due to restricted budget, it is rewarding to employ an extreme sampling design in the sense that there can be immense cost reductions and qualitatively similar power as in the full data analysis.

## Introduction

Despite the successful application of genome-wide association studies (GWAS) in hundreds of traits, the genetic variants discovered so far explain only a small proportion of the total heritability of complex traits^1,2^. As a result, considerable attention in recent years has turned to extremely large sample size array- or sequencing-based genome-scale association studies in the search for more undiscovered causal variants^3^. But it is notoriously expensive to genotype a large number of individuals, especially in sequencing studies. In addition, there is the need to adjust for multiple testing. The Bonferroni-corrected p-value, the significance threshold set to 0.05 divided by the total number of SNPs analyzed (e.g., p = 1 × 10^−7^ for 500,000 (500 K) SNPs), is generally applied to control the family-wise error rate. Therefore, low statistical power is a major concern in genetic association studies.

Statistical power is the probability to reject a null hypothesis (H_0_) while the alternative hypothesis (H_A_) is true. To improve power, for a quantitative trait, it has been proposed that one cost-effective strategy for enriching the presence, or absence, of a causal allele in a sample and reducing the cost of variant calling is to only take extreme observations of the trait distribution and carry out a case-control study instead of a regular QTL mapping study. In a GWAS study of hypertension, Padmanabhan *et al*. used an extreme case-control strategy by taking the top 2% of the blood pressure distribution as cases and the lower 9.2% of the distribution as controls, and discovered a SNP in the uromodulin gene to be associated with hypertension^4^. Other researchers compared different thresholds in the distribution of body mass index, height and waist-to-hip ratio for anthropometric traits and this way identified 11 novel loci^5^. Schork *et al*. focused on theoretical calculations for the power of extreme sampling under different scenarios of LD strength and heritability^6^. For common variants, Huang and Lin (2007) proposed testing for associations between extreme continuous phenotypes and variants using the maximum likelihood method assuming a truncated normal distribution for extreme phenotype^7^. Uemoto *et al*. (2011) accounted for pedigree structure using selective genotyping strategy, and identified 32 loci associated with oleic acid (C18:1) in the intramuscular fat of the trapezius muscles in Japanese Black cattle^8^. Recently, Barnett *et al*. extend the extreme case-control methods to identify rare variants in Sequencing Association Studies^9^.

In this article, we investigate in a straightforward way the power of using extreme phenotype samples defined by different thresholds, for which unbalanced selections of cases and controls are rarely discussed previously. We simulate a wide range of scenarios under dominant, recessive, and multiplicative models to identify situations where extreme selection strategies should profitably be employed. The enhancement in power is also illustrated through two real data application: the methadone doses (MD) in former heroin addicts undergoing methadone maintenance treatment (MMT) in Israel^10^, and the yearling weight in Korean native beef cattle^11^. Our observations offer a practical guide for researchers to choose an appropriate threshold in defining cases and controls.

## Materials and Methods

### Definitions and models

Define a QTL locus with two alleles, A and B, with the B allele conferring risk. Under a dominant model, let the mean phenotypic value for individuals with genotype AA be 1 and the phenotypic values for individuals with AB and BB be δ, δ > 0. Similarly, under a recessive model, the mean phenotypic values for AA, AB and BB are 1, 1, and δ, respectively. Under a multiplicative model, the means should be 1, δ and δ^2^. The genotype with mean value 1 is considered the reference genotype. Individuals with trait value y less than a threshold T_1_ are defined as control subjects and individuals with y > T_2_ are case subjects. We consider selecting individuals from the tails of the quantitative distribution so that we hopefully increase the proportion of sampled individuals with and without the risk allele in cases and controls, respectively, compared to the proportions in dichotomous disease groups with the threshold equal to the median of y. Note that the controls may not be controls in a strict sense in certain scenarios. From clinical point of view, both extremes may be interesting and can be defined as “cases” in case control analysis. In such situation, the definition of cases and controls can be tricky. In this study, controls refer to relatively healthy individuals obtained from a quantitative trait, or those that are cheaper and easier to recruit.

Among various tools employed for determining significant markers, Fisher’s exact test is classical and largely acceptable^12^, and thus often used to be compared with^13^. Therefore, we choose the classical Fisher’s exact test to assess the association between the marker and disease status. Table 1 displays a simple 2 × 3 contingency table from which a Fisher’s exact p-value can be derived.

**Table 1:** Illustration defining numbers of individuals with different genotypes from the two ends of the trait distribution.

	AA	AB	BB	Total
Upper tail	U_AA_	U_AB_	U_BB_	U
Lower tail	L_AA_	L_AB_	L_BB_	L
Total	Y_AA_	Y_AB_	Y_BB_	U + L

Under the null hypothesis of no association in the dominant model and given the observed margins, the probability of an observed table is hypergeometric,

$${\rm{p}}({U}_{AA})=\frac{(\begin{array}{c}U\\ {U}_{AA}\end{array})(\begin{array}{c}L\\ {L}_{AA}\end{array})}{(\begin{array}{c}U+L\\ {Y}_{AA}\end{array})},$$

with the symbols being defined in Table 1. Fisher’s exact p-value is then calculated as the probability of observing this or more extreme tables. Similarly, Fisher’s exact test can be applied under the recessive model and extended to be applied under the multiplicative model.

In addition to Fisher’s exact, we employ XP-GWAS to identify trait-associated variants. The method is developed in particular for individuals that have extreme phenotypes^14^.

### Simulation procedures and dataset

To provide a practical guide for choosing an appropriate threshold in selecting cases and controls, we conduct extensive simulations to study the relationship between power, fraction of selected samples, effect size and minor allele frequency (MAF). For a sample size of n = 300, we first generate genotype data under the assumption of HWE. Let the frequency of the minor allele B be p, so the frequency of the major allele A becomes q = 1 − p. The three respective genotype frequencies in the population are then p^2^, 2pq and q^2^. To generate a genotype, two alleles are drawn independently from a binomial distribution B(n, p). After SNP genotypes have been generated, we simulate phenotype data for individuals with a specific genotype. Phenotype data are generated from normal distributions with different specifications of mean values between genotypes under the various genetic models (See section Definitions and Models). We repeat this process 10,000 times.

Analysis of real data can provide more relevant information than simulation. In analysis of real data, we first describe our analysis of a dataset on dose required for effective MMT^6^, followed by extensive simulations comparing the performance of the QTL and threshold-defined case-control selection approach. For the quoted dataset, investigators collected blood samples from former heroin addicts who were all stabilized with methadone. One hundred and ten SNPs from eleven genes encoding potential pharmacodynamic factors of methadone were analyzed. The study was approved by the Helsinki Committee of the Tel-Aviv Sourasky Medical Center and The Rockefeller University Hospital institutional review board, and all subjects signed informed consent for genetic studies (additional information may be found in ref.^10^).

The other data set comes from the genome-wide association study for yearling weight in Korean native beef cattle^11^. The data set comprised 486 Hanwoo steers that were born between spring of 2005 and fall of 2007 in Hanwoo Improvement Center (HIC) of Nonghyup in Seosan, Korea. Yearling weights of the steers were measured in the HIC and the DNAs of the steers were provided by the HIC laboratory under the approval of the Hanwoo research committee of the Technology Development Program for Agriculture and Forestry, Ministry of Agriculture, Forestry and Fishers, Republic of Korea in 2010. The steers were genotyped with the 35,968 SNPs that were embedded in the Illumina bovine SNP 50 K beadchip and yearling weight traits were measured for the steers. Ethics committee approval for treatment of animals was not required, as all the blood samples and measurement of the trait were taken by veterinarians for routine purposes in HIC.

### Software

The program used to simulate and analyze the data has been written in the R statistical programming language (http://www.r-project.org). Code for simulation and evaluating power of the methods is available from the Supplementary Code.

## Results

### Simulations

**Figure 1:**
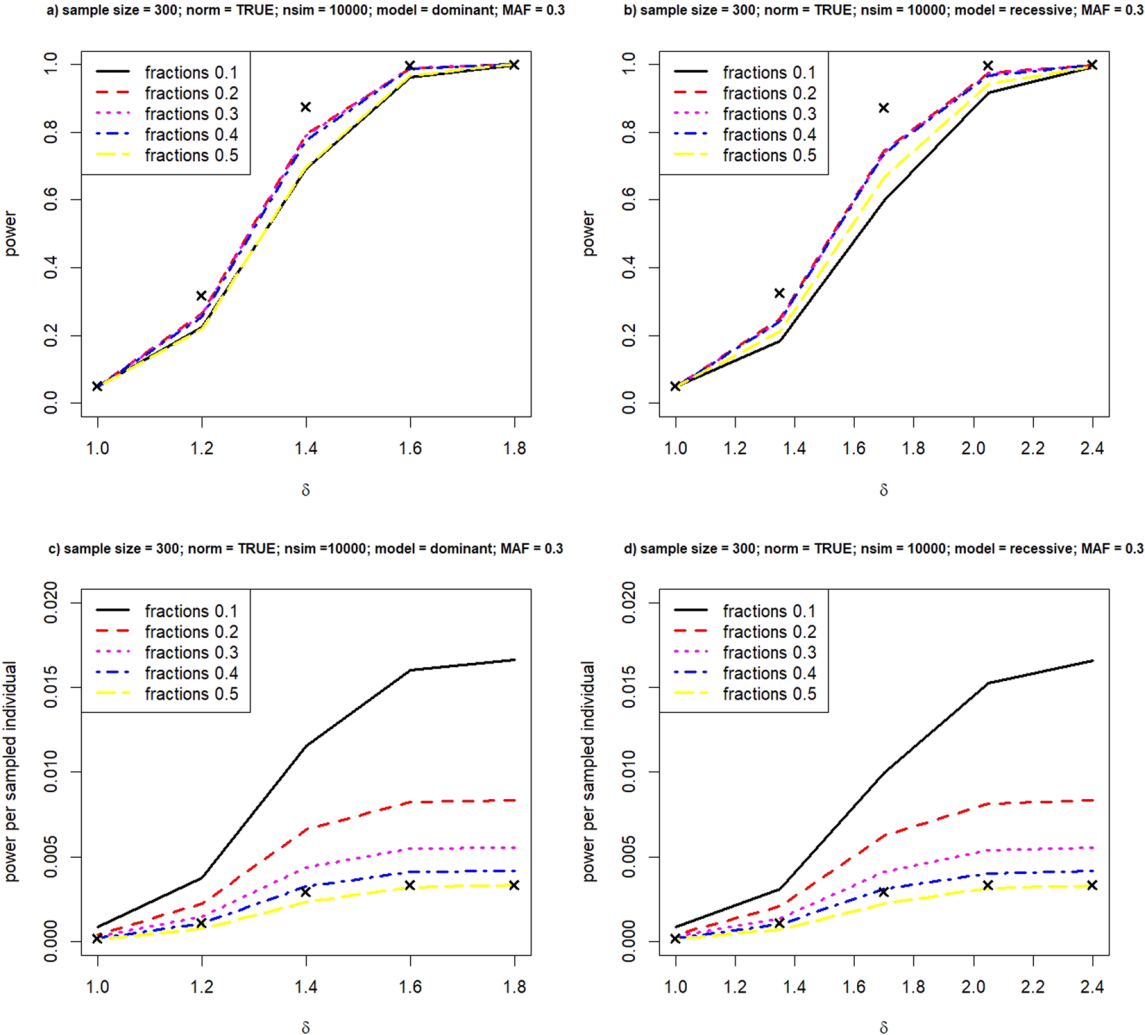
Power of extreme study design under the dominant and recessive model. Relationship between power and various fractions in selecting cases and controls, under the dominant model (left panel) and the recessive model (right panel). Crosses represent achieved power when we analyze the phenotype value quantitatively.

**Figure 2:**
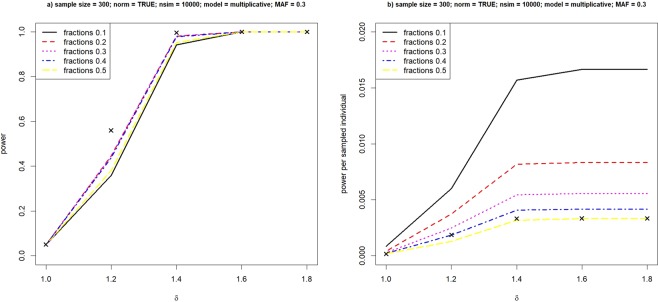
Power of extreme study design under the multiplicative model. Relationship between power and various fractions in selecting cases and controls, under the multiplicative model. Crosses represent achieved power when we analyze the phenotype value quantitatively.

We begin with a simulation where the risk allele has a moderate frequency of p = 0.3. We would like to investigate the impact on power when we select cases and controls from different fractions from the two ends of the phenotype distribution. In Figs 1 and 2, power overall and power per sampled individual is plotted against the mean trait value in the non-reference genotype. A fraction of 0.1 means that we select 10% of all individuals from the upper tail of the phenotype distribution as cases and an equal number of controls from the lower tail. When δ = 1, there is no difference of phenotype means between genotypes, and we expect the type I error rate to be 5% if it is specified at 0.05. Sometimes the type I error rate can deviate from 5% due to various reasons, for example, insufficient sample size. In order to achieve a fair comparison, we apply a calibrated cutoff to obtain a constant significance level for association tests so that the type I error rate is always 5% under the null hypothesis of δ = 1 regardless of the percentage of cases and controls selected from the full samples. We can calculate the type I error rate for a range of cutoff values. We select the cutoff such that the type I error rate is exactly 5% as the calibrated cutoff.

Crosses in the figures represent yielded power when we treat the phenotype value quantitatively as it is. From Fig. 1a,b), we clearly see that it is not wise to select too few individuals with extreme phenotypes, nor simply dichotomizing all samples by the median value. It is difficult to outperform ANOVA in terms of total power. But sampling extreme phenotypes performs qualitatively similar to the full quantitative analysis, especially when the difference of mean values between the genotypes becomes large. For examples, at δ ≥ 2 under both the dominant and recessive model, using extreme individuals would be as powerful as the conventional quantitative analysis. It implies that there can be immense cost reductions and researchers would save as much as 60% cost in genotyping, when cases and controls are selected from the top and bottom 20% of the phenotype values. Power per sampled individual is presented in Figure c) and d), where the difference between varied sampling fractions become much obvious. When the true model is multiplicative, Fig. 2 reveals a similar pattern in comparing the power of quantitative and binary analyses. Therefore, in the following simulation, we do not focus on the multiplicative model.

**Figure 3:**
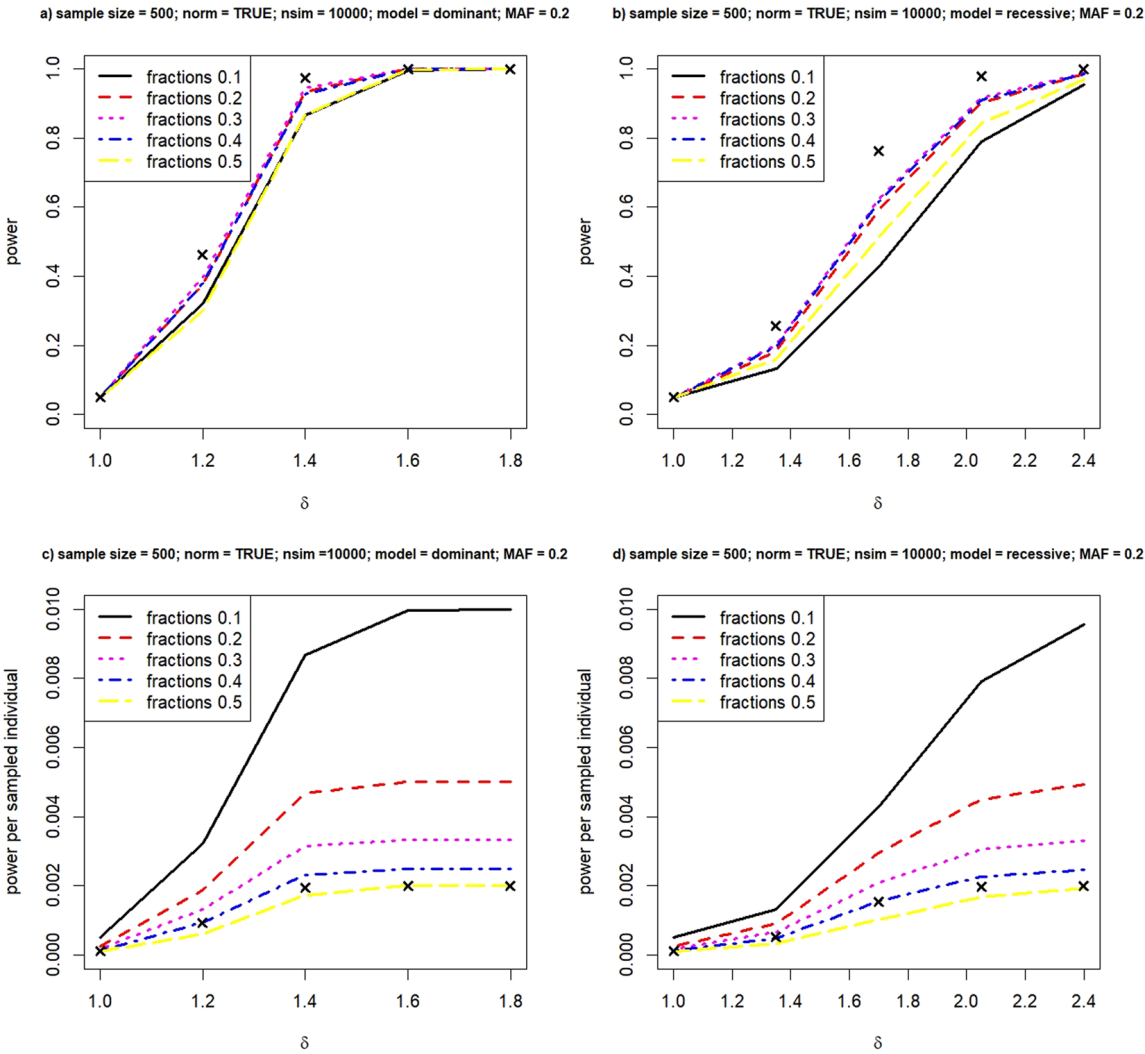
Power versus fractions in selecting equal number of cases and controls. Relationship between power and various fractions in selecting cases and controls, under the dominant model (left panel) and the recessive model (right panel). Crosses represent achieved power when we analyze the phenotype value quantitatively.

Since sampling phenotypic extremes is motivated by increasing the probability of the presence of a risk allele in a sample, we would imagine that when MAF drops it is more difficult to sample individuals with the risk genotypes, especially under a recessive model. Figure 3 is generated in the same way as Fig. 1 except that MAF is reduced to 0.2. We also increased the sample size from 300 to 500 to avoid the situation that individuals with two risk alleles do not occur in simulated samples. Again, we see an obvious advantage of sampling extremes in terms of cost reduction. Under the dominant model, analyzing data as a continuous or binary variable is comparable in the total power, even when δ is small. The effect of dichotomizing under the multiplicative model is documented in Supplementary Fig. S2.

**Figure 4:**
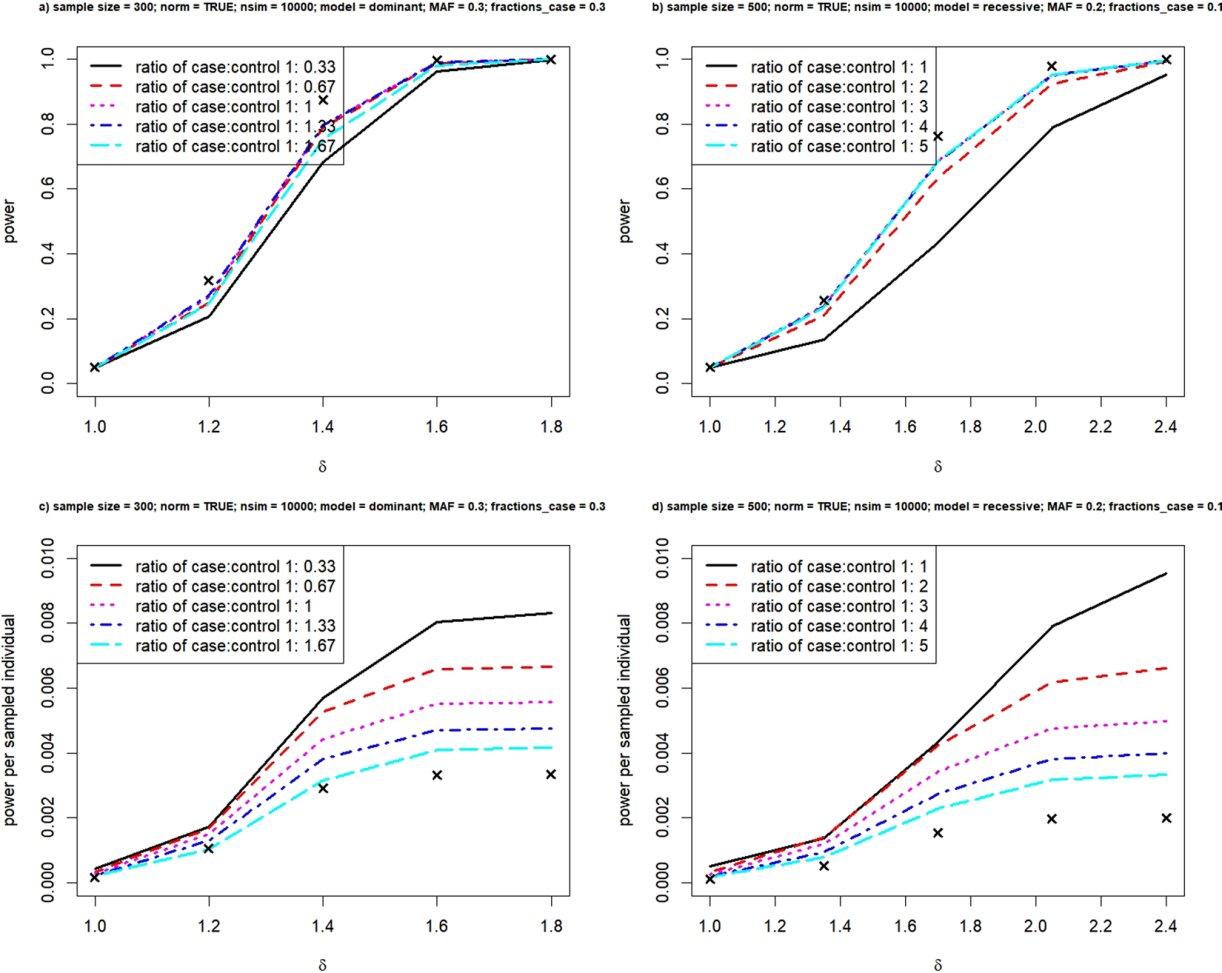
Power versus fractions in selecting cases and controls. Relationship between power and various unequal fractions in selecting cases and controls, under the dominant model (left panel) and the recessive model (right panel). Crosses represent achieved power when we analyze the phenotype value quantitatively.

In the previous simulations, equal numbers of cases and controls are selected. Generally, increasing number of controls per case results in greater power^15^. Increase in power, however, starts to level out when the ratio of cases and controls is beyond 1:4. It is natural to ask whether the ratio of cases and controls has an impact on power under extreme sampling. We first fix the fraction at 0.1 in selecting cases and vary the fraction in controls from 0.1 to 0.5; then we fix the fraction at 0.2 in cases and vary the fraction in controls, and so on. Figure 4 presents the simulation results under two settings. The ones on the left set MAF = 0.3 and under the dominant model where the number of individuals in the reference genotype group and risk group almost match, thus the phenotype distribution is approximately symmetric since it can be considered as a balanced mixture of two normal distributions. The ones on the right set MAF = 0.2 under the recessive model.

A series of five plots are generated under each setting using unequal fractions in cases and controls. Figure 4 only presents one sets. The rest of the plots can be found in Supplementary Figs S3 and S4. Figure 4a indicates that under the dominant model with MAF = 0.3 and fraction of cases = 0.3, it is not wise to have too few controls. Therefore, the most economic selection would be a case-control ratio of 1:0.67 (Fig. 4c). While under the recessive model with MAF = 0.2 and fraction of cases = 0.1, an optimal ratio of cases and controls is 1:2 or 1:3.

We show the comparison between extreme sampling, random sampling of the same size with extreme sampling and XP-GWAS in Table 2. Extreme sampling outperforms the other two methods when sampling fraction < 0.4 under dominant model. By random sampling, it means that we draw samples randomly from the whole eligible range of the quantitative phenotype distribution, instead of the extreme ends. Given the sample size and cost of random sampling is the same as in the extreme sample design, the latter is more powerful especially when budget is limited. Therefore, a small fraction should be sampled from the ends of phenotypic distribution.

**Table 2:** Comparisons between extreme sampling, random sampling and XP-GWAS.

δ	Fractions	Full data			Extreme sampling			Random sampling			XP-GWAS		
		Dominant	Recessive	Multiplicative	Dominant	Recessive	Multiplicative	Dominant	Recessive	Multiplicative	Dominant	Recessive	Multiplicative
1	0.1	0.05	0.05	0.05	0.0501	0.0515	0.0501	0.0500	0.0504	0.0500			
	0.2				0.0500	0.0505	0.0500	0.0500	0.0500	0.0500			
	0.3				0.0500	0.0500	0.0500	0.0501	0.0500	0.0501			
	0.4				0.0500	0.0512	0.0500	0.0500	0.0510	0.0507			
	0.5				0.0500	0.0508	0.0500	0.0505	0.0511	0.0500			
1.1	0.1	0.1105	0.07	0.1629	0.0923	0.0580	0.1178	0.0591	0.0518	0.0688	0.0520	0.0410	0.0657
	0.2				0.1013	0.0655	0.1421	0.0749	0.0585	0.0935	0.0606	0.0500	0.0806
	0.3				0.0966	0.0611	0.1355	0.0839	0.0617	0.1142	0.0639	0.0535	0.0844
	0.4				0.0947	0.0690	0.1305	0.0972	0.0688	0.1402			
	0.5				0.0906	0.0635	0.1205	0.1058	0.0668	0.1553			
1.2	0.1	0.3188	0.1361	0.5604	0.2248	0.0883	0.3597	0.0964	0.0643	0.1371	0.0826	0.0522	0.1502
	0.2				0.2654	0.1138	0.4473	0.1495	0.0809	0.2468	0.0961	0.0613	0.1977
	0.3				0.2609	0.1033	0.4457	0.2023	0.0973	0.3625	0.1069	0.0665	0.2061
	0.4				0.2556	0.1098	0.4378	0.2686	0.1156	0.4741			
	0.5				0.2197	0.1008	0.3816	0.3091	0.1272	0.5507			
1.3	0.1	0.6297	0.2467	0.915	0.4398	0.1468	0.7124	0.1546	0.0827	0.2712	0.1349	0.0673	0.3131
	0.2				0.5299	0.1946	0.8309	0.2837	0.1182	0.5206	0.167	0.0831	0.4136
	0.3				0.5240	0.1905	0.8283	0.4198	0.1593	0.7177	0.1823	0.088	0.4253
	0.4				0.5175	0.1883	0.8190	0.5428	0.2070	0.8442			
	0.5				0.4465	0.1665	0.7473	0.6287	0.2403	0.9136			
1.4	0.1	0.8755	0.4107	0.9961	0.6940	0.2283	0.9414	0.2450	0.1084	0.4777	0.2095	0.0899	
	0.2				0.7951	0.3155	0.9816	0.4741	0.1794	0.8046	0.2639	0.1148	0.6773
	0.3				0.7894	0.3079	0.9807	0.6667	0.2574	0.9420	0.2843	0.1200	0.6892
	0.4				0.7775	0.3042	0.978	0.7987	0.3432	0.9858			
	0.5				0.6981	0.2651	0.9514	0.8777	0.4012	0.9956			

### Real data analysis

We assessed the extreme case-control strategy by application in the two relevant datasets. In the hope of increasing power to detect marker-phenotype association, we tested each SNP for association between genotypes AA, AB, BB versus trait in extreme samples using a univariate logistic regression model. The trait values, trait, are ordered by magnitude and individuals with T_1_ < trait < T_2_ are removed from the analysis. Individuals with trait value ≤ T_1_ are labeled controls and those with trait value ≥ T_2_ are labeled cases. T_1_ and T_2_ can be chosen from the percentile values in the lower and upper tail of the trait distribution.

### MD study

The enhancement in power is also illustrated through a real data application to analysis of methadone doses (MD) in former heroin addicts undergoing methadone maintenance treatment (MMT) in Israel^6^. We have 227 individuals in this study. The distribution of MD is presented in Supplementary Fig. S1. It looks rather normal so that no transformation appears necessary. In all the samples, the genomic inflation factor is equal to one, indicating no evidence for population stratification, perhaps because of the small sample size. Deviations from Hardy-Weinberg Equilibrium (HWE) for all 110 SNPs are also not significant when adjusted for multiple testing.

We first examine the association between SNPs and methadone doses using the traditional QTL analysis. There are five statistically significant SNPs (p-value <= 0.05). The smallest unadjusted p-value from ANOVA is 0.027 (rs4358872, MAF = 0.45, effect size = 11.24). Let T_1_ = t-percentile and T2 = (1-tpercentile), meaning we take equal fractions of individuals from the tails of the distribution. Next, we vary T1 from the 10th to 50th percentile. The median number of significant SNPs is 6.5. When T1 = 32^th^ percentile, the smallest p-value equals to 0.0026 for SNP rs10835210. SNP rs4358872 resides in intron 21 of gene NTRK2 (Neurotrophic Receptor Tyrosine Kinase 2) and rs10835210 is in intron 8 of gene BDNF (Brain Derived Neurotrophic Factor). These two SNPs have also been shown to be associated with MD^10^.

Next, we vary T1 between the 10th and 50th percentile and also T2 between the 50th and 100th percentile, because it is known that increasing the control-to-case ratio beyond 1:1 may result in higher power^15^. Some of the results are indeed better than when we select equal fractions of extreme individuals. The number of significant SNPs and the smallest p-value is summarized in Table 3.

**Table 3:** Individuals with MD > T_2_ and MD < T_1_ are selected as cases and controls, respectively.

Controls (MD < T_1_)	Cases (MD > T_2_)				
	90^th^	80^th^	70^th^	60^th^	50^th^
**(a) List of number of significant SNPs from the univariate logistic regression model**.					
10^th^	2	3	4	3	3
20^th^	4	10	13	5	7
30^th^	7	9	16	4	7
40^th^	7	8	11	4	10
50^th^	6	8	15	5	10
**(b) List of the smallest p-values from the univariate logistic regression model**.					
10^th^	0.014	0.002	0.001	0.002	0.001
20^th^	0.006	0.008	0.016	0.004	0.010
30^th^	0.005	0.017	0.010	0.009	0.024
40^th^	0.006	0.018	0.005	0.012	0.020
50^th^	0.003	0.014	0.008	0.006	0.009

### Yearling weight study

Weight traits are considered the most economically important production traits in beef cattle. The animals are weighed at predefined times to comply with the respective breeding scheme, where common measurements are taken at 12 months, yearling weight. A genome-wide association study for yearling weight in Korean native beef cattle has been reported by co-authors Yi Li *et al*.^11^. Among the 54,001 SNPs in the Illumina bovine beadchip array, 35,968 SNPs were available for tests. The 486 steers in this study represent a full sample (all samples were genotyped) and the trait yearling weight can be assumed normally distributed in the population. The corrected phenotypes are estimated by the fixed effect (farm, year and season of birth), covariate (yearling age) and random additive polygenic effect. We therefore perform test using the high and low corrected phenotypic tails of the corrected phenotypic distribution in the population.

We first examine the association between SNPs and yearling weight using the traditional QTL analysis. We assume a conservative threshold value by using the Bonferroni correction to reduce type I error (p = 1.39 × 10^−6^ for 35,968 SNPs). The smallest unadjusted p-value from ANOVA is 1.09 × 10^−5^ (ARS-BFGL-NGS-105590, MAF = 0.28, effect size = 4.45), which is not significant after Bonferroni correction. Then, we tested each SNP for association between genotypes AA, AB, BB versus yearling weight in extreme samples using the Fisher’s exact test. Let T_1_ = t-percentile and T2 = (1-tpercentile), meaning we take equal fractions of individuals from the tails of the distribution. When we vary T_1_ from the 10^th^ to 50^th^ percentile, there is one statistically significant SNP, Hapmap36817-SCAFFOLD245829_8774 (MAF = 0.23, effect size = 0.40). The SNP has a p-value of 1.77 × 10^−7^, obtained with T_1_ = 13^th^ percentile. The SNP resides in gene *FAT2* (FAT Atypical Cadherin 2) and SNP Hapmap36817-SCAFFOLD245829_8774 resides in gene *GAS2* (Growth Arrest Specific 2). These two SNPs have also been shown to be associated with yearling weight^10^. Next, when we vary T1 between the 10th and 50th percentile and also T2 between the 50^th^ and 100^th^ percentile, the number of significant SNP remains one with multiple testing correction. If we relaxed the threshold of statistical significance to 10^−4^, we can detect two SNPs using full data, while 3–5 SNPs are detected under the extreme sampling design. The p-value for ARS-BFGL-NGS-105590 decreases to 6.07 × 10^−7^ (MAF = 0.28, effect size = 0.88) with T1 = 26^th^ and T2 = 85^th^. The smallest p-value of 3.00 × 10^−8^ is obtained for Hapmap36817-SCAFFOLD245829_8774 (MAF = 0.22, effect size = 0.42) when T1 = 13^th^ and T2 = 80^th^, in other words, the control-to-case ratio is approximately 1:2. It shows that similar results as for full data can be obtained using extreme sampling with only a fraction of the data.

## Discussion

The cost of large-scale molecular studies is still relatively expensive and in some animal studies, it even has to kill cattle in order to measure the phenotypes. Therefore, researchers are attracted by potential gains in using extreme samples of population. However, practical guidance has not been well established. To provide some insights for large-scale association studies, we investigate the power of sampling the extremes and establish proper thresholds to select cases and controls with the changes of MAF and effect size. We show that when effect size is relatively large, it is beneficial to carry out an extreme sampling study. We confirm through extensive simulations that selecting between 20% and 40% of cases and controls, respectively, from the tails of the distribution could be as powerful as the full data analysis. Conventionally, adding more controls would yield more statistical power, but this effect is negligible above 4:1^16,17^. Here the most optimal case-control ratio is not 1:5 or 1:4, because it would relax the threshold too much in selecting controls such that many individuals with risk genotype are mixed into the control samples.

To avoid the multiple testing problem in searching for the threshold with the smallest p-value when the underlying genetic model is unknown, we would recommend researchers to go with the worst case scenario to avoid the problem of low power in detecting the associated risk allele. Worst case scenario refers to a genetic model where a relatively bigger sample size is required compared to the other genetic models, among dominant, recessive and multiplicative.

We demonstrate the application of the design through real data analyses. Note that although we arbitrarily define former heroin addicted individuals who needed extremely high MD in methadone maintenance treatment as cases, we should be aware that individuals with very low MD are not clinically healthy controls. It may partially explain that the lower/lowest p-value is observed when the number of cases is more than that of controls while according to the simulations, generally it is more powerful when the number of controls is more than that of case. It would be interesting to explore the biological mechanisms and factors that lead certain individuals to require only a very low MD in treatment. The idea of increasing the number of controls to increase power may come from situations in which the cost of obtaining controls is lower than the cost of obtaining cases, or in which cases may be difficult to find, while healthy controls are more easily available. If the definition of cases and controls is arbitrary, the oversampling idea should be applicable to both tails of the phenotypic distribution, or, consider oversampling the tail where it is much easier and cheaper to recruit individuals. When the total sample size is restricted due to cost and it is equally expensive to obtain cases and controls, the most powerful case-to-control ratio would be 1:1 [Jewell, 2004^18^] and no need to consider unbalanced case control design.

In the real data demonstrations, the detected SNPs might be unstable and should be interpreted cautiously, since they are derived from a small sample size. The smallest p-values may correspond to different SNPs with different parameters, for example, with different genetic models. Nevertheless, it is noted that the SNPs identified in the real data analysis have been demonstrated to be related to the phenotype in literature^10^.

The study has its limitations. Our conclusions are derived from a simulation study of limited scenarios. In the simulation, it is assumed that the minor allele increases the risk. Another scenario may be that the direction of effects of the minor allele is opposite or protective. But it would not change the conclusion of extreme sampling design being cost-effective, because the definition of cases and controls in quantitative traits can be arbitrary.

Note that we would not recommend practitioners to blindly use extreme sampling approaches for power improvement when full data is available, but rather to propose an alternative way of study design before any samples are collected.

## Conclusions

In practice, it is cost-effective to employ an extreme sampling design, which achieves similar power as the QTL analysis in most situations except when effect size is relatively small. To maximize power, it may worth to explore the choices of an unbalanced proportion in selecting cases and controls.

## Supplementary information

Supplementary Information

## Data Availability

The datasets used and analyzed during the current study are available on reasonable request.

## References

[1] Manolio TA (2009). Finding the missing heritability of complex diseases. Nature.

[2] Levy D (2009). Genome-wide association study of blood pressure and hypertension. Nat Genet.

[3] McCarthy MI (2008). Genome-wide association studies for complex traits: consensus, uncertainty and challenges. Nat Rev Genet.

[4] Padmanabhan S (2010). Genome-wide association study of blood pressure extremes identifies variant near UMOD associated with hypertension. PLoS Genet.

[5] Berndt SI (2013). Genome-wide meta-analysis identifies 11 new loci for anthropometric traits and provides insights into genetic architecture. Nat Genet.

[6] Schork NJ, Nath SK, Fallin D, Chakravarti A (2000). Linkage disequilibrium analysis of biallelic DNA markers, human quantitative trait loci, and threshold-defined case and control subjects. Am J Hum Genet.

[7] Huang BE, Lin DY (2007). Efficient association mapping of quantitative trait loci with selective genotyping. Am J Hum Genet.

[8] Uemoto Y (2011). Whole-genome association study for fatty acid composition of oleic acid in Japanese Black cattle. Anim Genet.

[9] Barnett IJ, Lee S, Lin XH (2013). Detecting Rare Variant Effects Using Extreme Phenotype Sampling in Sequencing Association Studies. Genet Epidemiol.

[10] Levran O (2013). Association of genetic variation in pharmacodynamic factors with methadone dose required for effective treatment of opioid addiction. Pharmacogenomics.

[11] Li Y, Gao Y, Kim YS, Iqbal A, Kim JJ (2017). A whole genome association study to detect additive and dominant single nucleotide polymorphisms for growth and carcass traits in Korean native cattle, Hanwoo. Asian-Australas J Anim Sci.

[12] Alghamdi, J. & Padmanabhan, S. *Handbook of Pharmacogenomics and Stratified Medicine* (Academic Press, 2014).

[13] Lunetta KL (2004). Screening large-scale association study data: exploiting interactions using random forests. BMC Genetics.

[14] Yang J (2015). Extreme-phenotype genome-wide association study (XP-GWAS): a method for identifying trait-associated variants by sequencing pools of individuals selected from a diversity panel. The Plant Journal.

[15] Ury HK (1975). Efficiency of case-control studies with multiple controls per case: continuous or dichotomous data. Biometrics.

[16] Cologne JB (2004). Improving the efficiency of nested case-control studies of interaction by selecting controls using counter matching on exposure. International Journal of Epidemiology.

[17] Hennessy S (1999). Factors influencing the optimal control-to-case ratio in matched case-control studies. American Journal of Epidemiology.

[18] Jewell, N. Statistics for Epidemiology (Boca Raton: Chapman & Hall/CRC, 2004).

