# Supplementary material for: Extreme sampling design in genetic association mapping of quantitative trait loci using balanced and unbalanced case-control samples
Source: Sci Rep. 2019 Oct 29;9:15504. doi: 10.1038/s41598-019-51790-w (PMC6820758; doi:10.1038/s41598-019-51790-w)
Supplement: Supplementary file 1 — Supplementary Information [file 41598_2019_51790_MOESM1_ESM.pdf]

Extreme sampling design in genetic association mapping of quantitative trait loci using balanced and unbalanced case-control samples

Yi Li[1], Orna Levran[2], JongJoo Kim[3], Tiejun Zhang[4,5], Xingdong Chen[6§] & Chen Suo[4,5§]

[1]School of Statistics, Shanxi University of Finance & Economics, Shanxi, China

[2]Laboratory of the Biology of Addictive Diseases, Rockefeller University, New York, US

[3]Department of Biotechnology, Yeungnam University, Gyeongsan, Korea

[4]Department of Epidemiology, School of Public Health, Fudan University, Shanghai, China

[5]Key Laboratory of Public Health Safety (Fudan University), Ministry of Education, China

[6]State Key Laboratory of Genetic Engineering and Collaborative Innovation Center for Genetics and Development, School of Life Sciences, Fudan University, Shanghai, China

[§]Corresponding author

XC: xingdongchen@fudan.edu.cn

CS: suochen@fudan.edu.cn

Supplementary figures

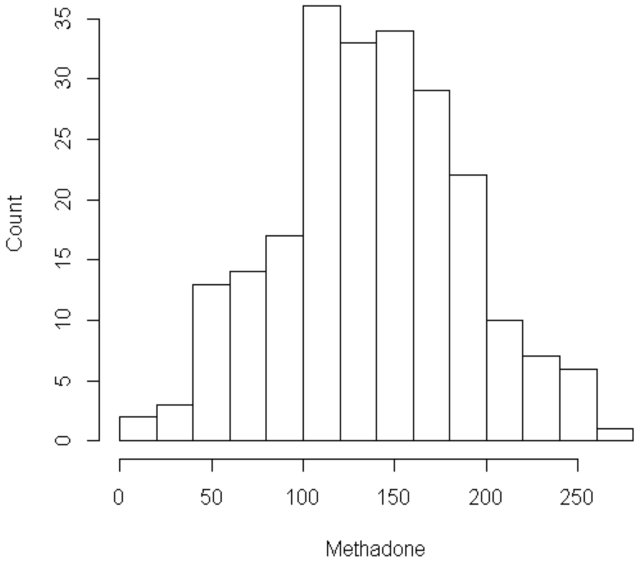

Figure S1 Histogram of methadone doses. The distribution of the methadone doses phenotype used in this study.

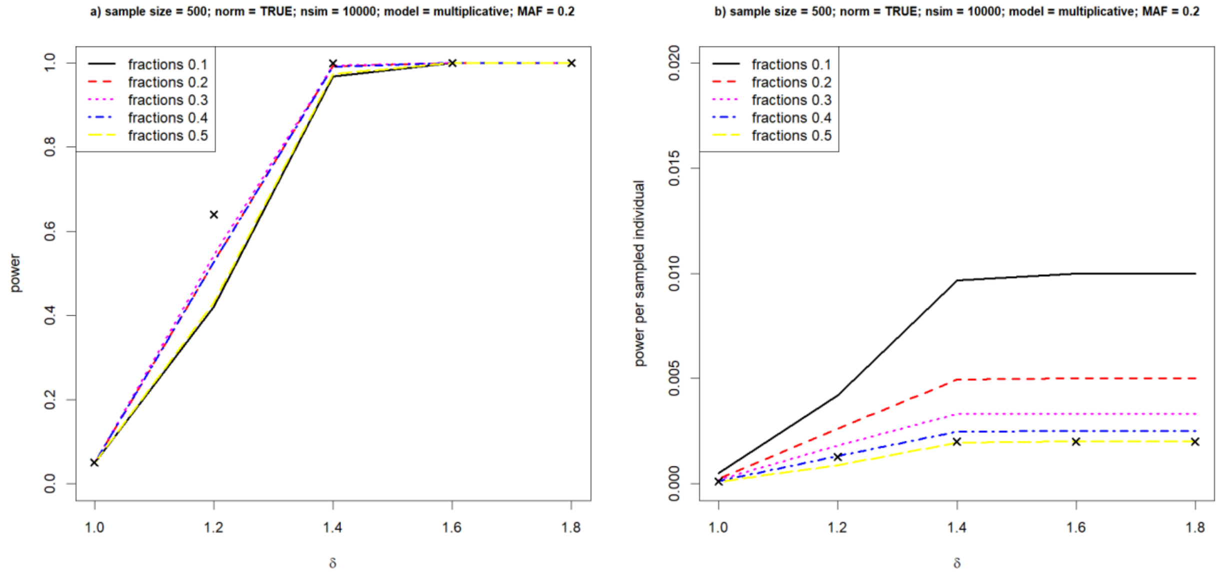

Figure S2 Relationship between power and various fractions in selecting cases and controls, under the multiplicative model.

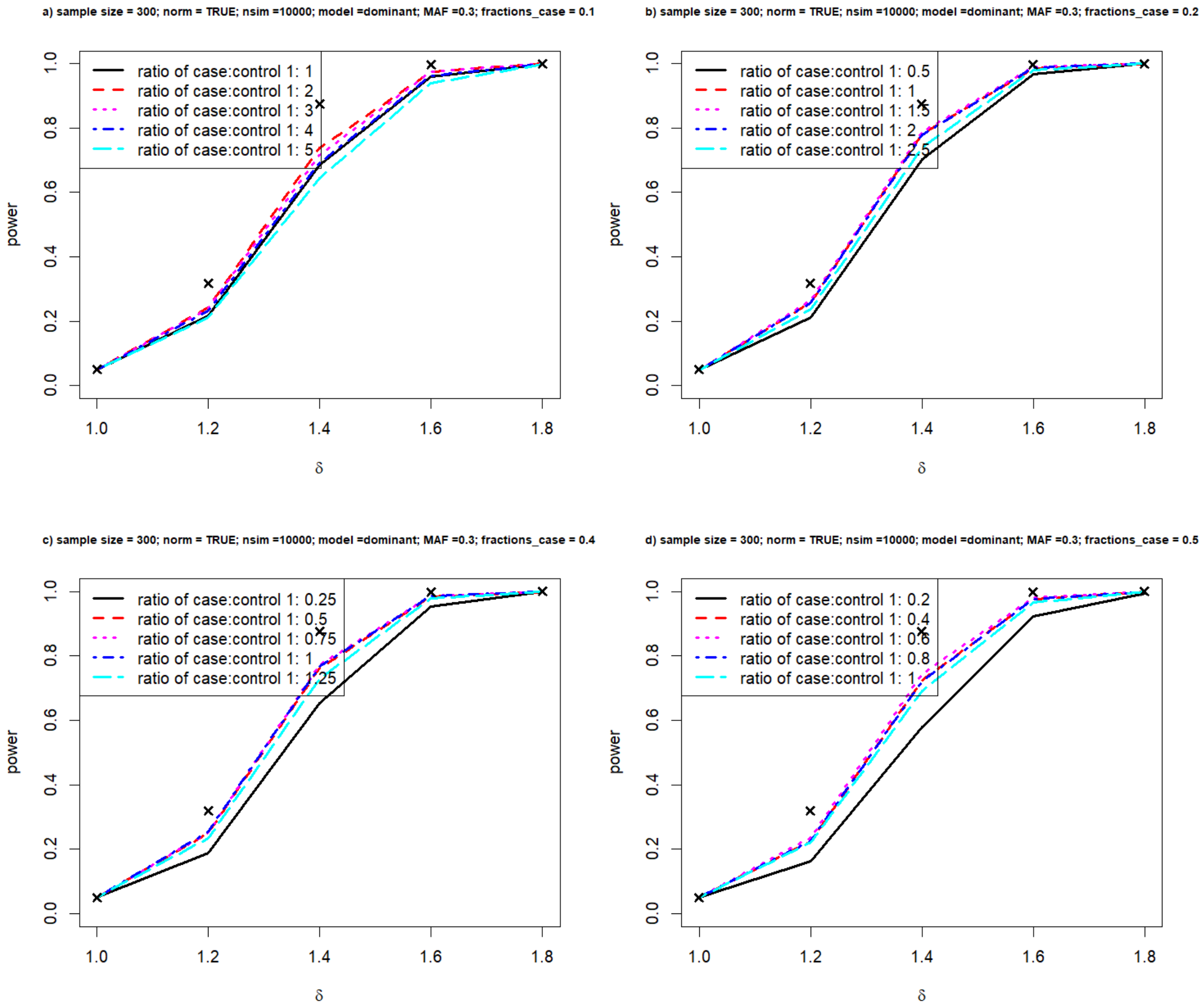
a) sample size = 300; norm = TRUE; nsim =10000; model =dominant; MAF =0.3; fractions_case = 0.1
ratio of case:control 1: 1
ratio of case:control 1: 2
ratio of case:control 1: 3
ratio of case:control 1: 4
ratio of case:control 1: 5
power
δ
b) sample size = 300; norm = TRUE; nsim =10000; model =dominant; MAF =0.3; fractions_case = 0.2
ratio of case:control 1: 0.5
ratio of case:control 1: 1
ratio of case:control 1: 1.5
ratio of case:control 1: 2
ratio of case:control 1: 2.5
power
δ
c) sample size = 300; norm = TRUE; nsim =10000; model =dominant; MAF =0.3; fractions_case = 0.4
ratio of case:control 1: 0.25
ratio of case:control 1: 0.5
ratio of case:control 1: 0.75
ratio of case:control 1: 1
ratio of case:control 1: 1.25
power
δ
d) sample size = 300; norm = TRUE; nsim =10000; model =dominant; MAF =0.3; fractions_case = 0.5
ratio of case:control 1: 0.2
ratio of case:control 1: 0.4
ratio of case:control 1: 0.6
ratio of case:control 1: 0.8
ratio of case:control 1: 1
power
δ

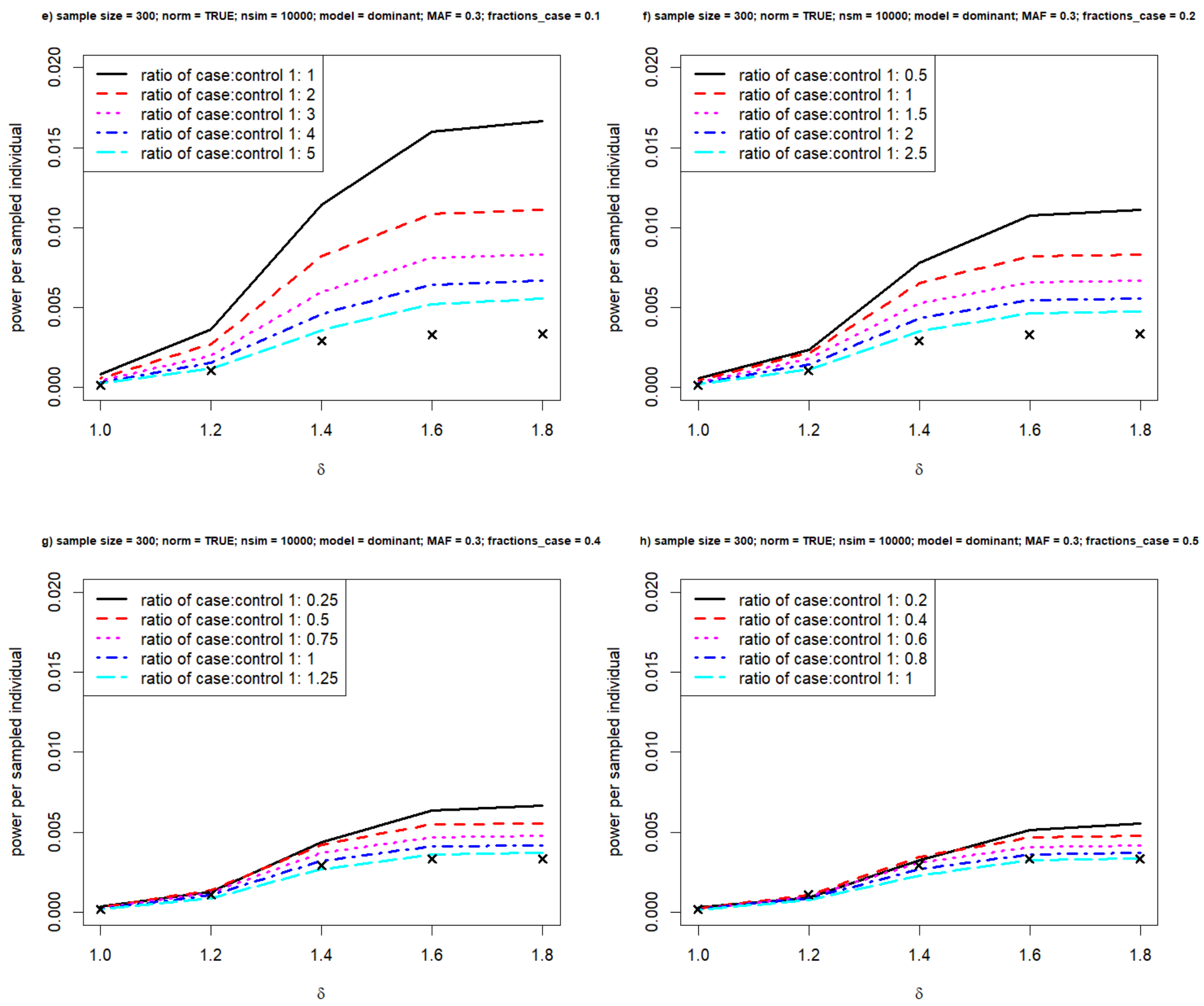

Figure S3 Relationship between power and various unequal fractions in selecting cases and controls, under the dominant model. The parameter settings are self-explanatory in the titles.

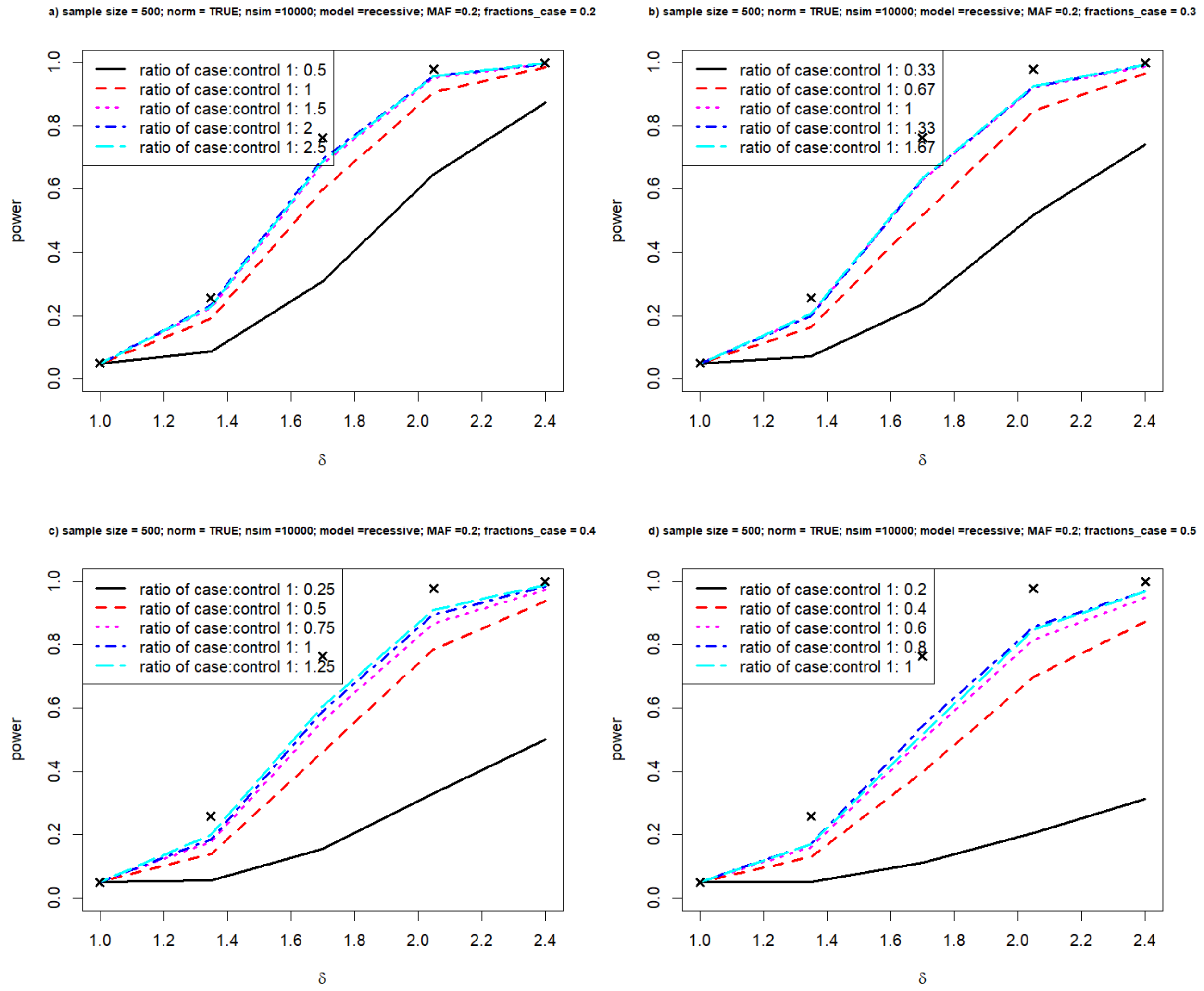
a) sample size = 500; norm = TRUE; nsim =10000; model =recessive; MAF =0.2; fractions_case = 0.2
ratio of case:control 1: 0.5
ratio of case:control 1: 1
ratio of case:control 1: 1.5
ratio of case:control 1: 2
ratio of case:control 1: 2.5
b) sample size = 500; norm = TRUE; nsim =10000; model =recessive; MAF =0.2; fractions_case = 0.3
ratio of case:control 1: 0.33
ratio of case:control 1: 0.67
ratio of case:control 1: 1
ratio of case:control 1: 1.33
ratio of case:control 1: 1.67
c) sample size = 500; norm = TRUE; nsim =10000; model =recessive; MAF =0.2; fractions_case = 0.4
ratio of case:control 1: 0.25
ratio of case:control 1: 0.5
ratio of case:control 1: 0.75
ratio of case:control 1: 1
ratio of case:control 1: 1.25
d) sample size = 500; norm = TRUE; nsim =10000; model =recessive; MAF =0.2; fractions_case = 0.5
ratio of case:control 1: 0.2
ratio of case:control 1: 0.4
ratio of case:control 1: 0.6
ratio of case:control 1: 0.8
ratio of case:control 1: 1
power
δ

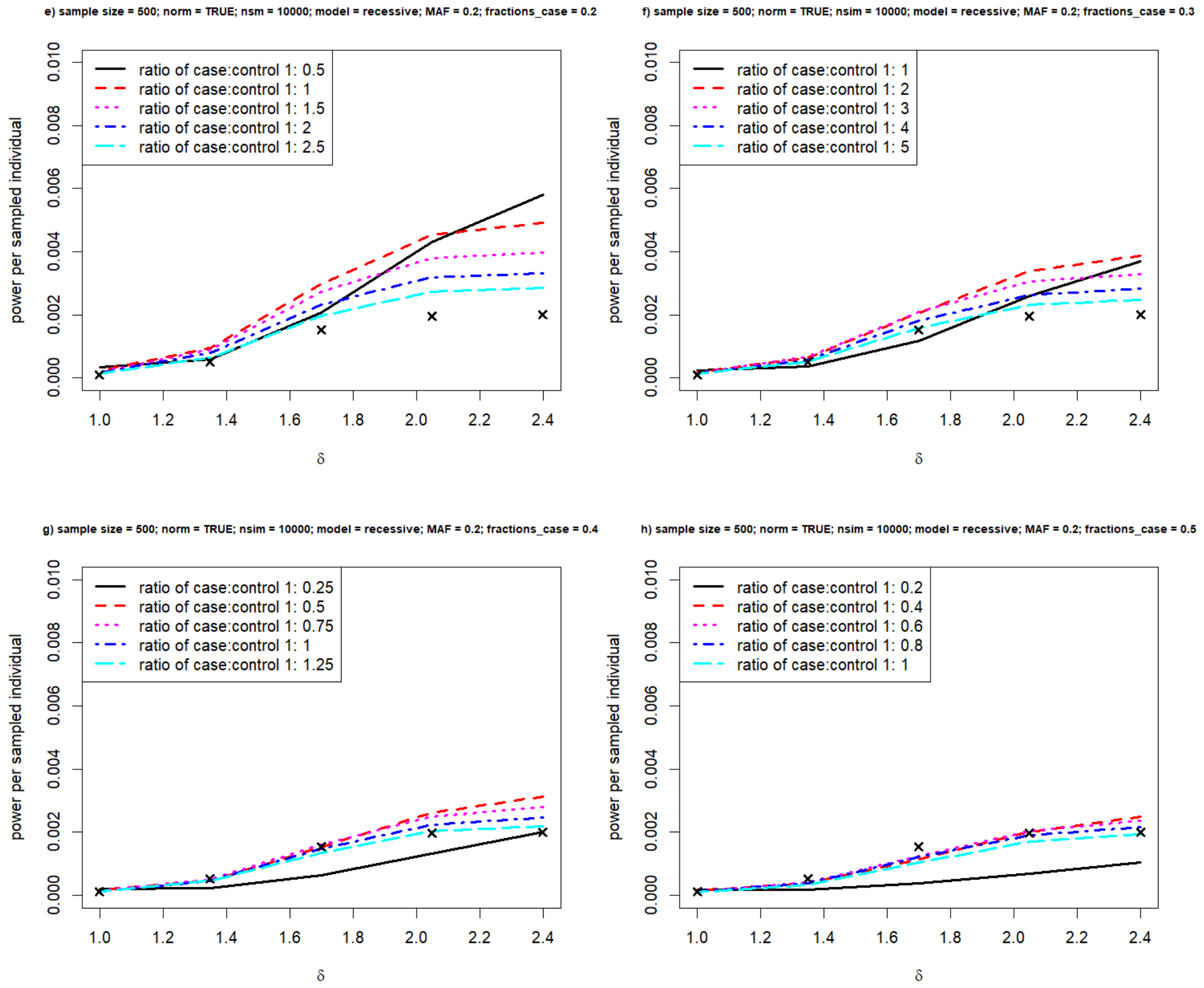

Figure S4 Relationship between power and various unequal fractions in selecting cases and controls, under the recessive model. The parameter settings are self-explanatory in the title.

Supplementary code

An R function to simulate a quantitative phenotype y and a snp x associated with y

Written by: Chen Suo

```
####The following is a description of parameters in the function simuXY####

##############################################################################

#number of simulations: nsim

#subjects = n, snps = m

#means in different genotype groups mu <- c(1*delta^2, 1*delta, 1)

#proportion in tails of phenotype distribution, p=0.25

#allele freq q1. E.g q1 = 0.3, so gene freq is about 0.49(00), 0.42(01) and 0.09(11)

#use p-value, or test statistics pval=FALSE

#a seed can be used to restore the random number generation in R: #set.seed(1611)


##vary proportions

simuXY <- function(nsim=100, n=300, n2=100, m=1, delta=1.2, mu=c(1*delta^2, 1*delta, 1), q1=0.3,
SD=1, p=seq(0.1, 0.5, length.out=10), pval=T, norm=T, seed=set.seed(1611), model=c("dominant",
"multiplicative", "recessive"),random_sampling=TRUE){


  ind.name <- sapply(c(1:n), function(x){paste("sample", x, sep="")})

  snp.name <- sapply(c(1:m), function(x){paste("snp", x, sep="")})

  sim.name <- sapply(c(1:nsim), function(x){paste("sim", x, sep="")})

  nf <- length(p)

  f.name <- sapply(c(1:nf), function(x){paste("f", round(x, 2), sep="")})
```

```
stats <- list()

tmax <- matrix(NA, ncol=nsim, nrow=length(p))

seed

stats[['pval']] <- stats[['Perm']] <- matrix(NA, ncol=nf, nrow=nsim, dimnames = list(sim.name, f.name))


for(sim in 1:nsim){


  for(pr in p){

    j <- which(p%in%pr)

    #for(pr2 in p){

    #j <- which(p%in%pr)




    #simulate X

    a1 <- sapply(c(1:m), function(x){rbinom(1, n=n, prob=q1)})

    a2 <- sapply(c(1:m), function(x){rbinom(1, n=n, prob=q1)})

    x <- a1+a2

    colnames(x) <- snp.name

    x <- x[order(x[,"snp1"]),]

    #we need to order such that the phenotype and genotype is corresponding

    if(is.vector(x))

      dim(x) <- list(n, 1)

    rownames(x) <- ind.name

    colnames(x) <- snp.name
```

```
#simulate Y
ni <- table(x[,"snp1"])
if(norm){
  if(model=='multiplicative'){
    y11 <- rnorm(n=ni[1], mean=mu[3], sd=SD)
    y12 <- rnorm(n=ni[2], mean=mu[2], sd=SD)
    y22 <- rnorm(n=ni[3], mean=mu[1], sd=SD)
  }
  if(model=='dominant'){
    y11 <- rnorm(n=ni[1], mean=mu[3], sd=SD)
    y12 <- rnorm(n=ni[2], mean=mu[2], sd=SD)
    y22 <- rnorm(n=ni[3], mean=mu[2], sd=SD)
  }
  if(model=='recessive'){
    y11 <- rnorm(n=ni[1], mean=mu[3], sd=SD)
    y12 <- rnorm(n=ni[2], mean=mu[3], sd=SD)
    y22 <- rnorm(n=ni[3], mean=mu[2], sd=SD)
  }
} else {
  if(model=='multiplicative'){
  #this would the same as norm=T, as it is only a monotone transformation.
  #Ordering of phenotype values does not change.
    y11 <- rnorm(n=ni[1], mean=exp(mu[3]), sd=SD)
    y12 <- exp(rnorm(n=ni[2], mean=mu[2], sd=SD))
    y22 <- exp(rnorm(n=ni[3], mean=mu[1], sd=SD))
```

```
  }
  if(model=='dominant'){
    y11 <- rnorm(n=ni[1], mean=exp(mu[3]), sd=SD)
    y12 <- exp(rnorm(n=ni[2], mean=mu[2], sd=SD))
    y22 <- exp(rnorm(n=ni[3], mean=mu[2], sd=SD))
  }
  if(model=='recessive'){
    y11 <- rnorm(n=ni[1], mean=exp(mu[3]), sd=SD)
    y12 <- rnorm(n=ni[2], mean=exp(mu[3]), sd=SD)
    y22 <- exp(rnorm(n=ni[3], mean=mu[2], sd=SD))
  }
 }

 y <- c(y11, y12, y22)
 names(y) <- ind.name

if(random_sampling == TRUE){
			####Random sampling################
			sub_n <- 2*pr*n
			ind.name.sample <- sample(ind.name,sub_n)
			y.sub1 <-y[ind.name.sample]
			x.sub1 <-x[ind.name.sample,]
			stats1 <- getm(y.sub1,x.sub1, p=1)
	}else{
				if(p==1){
```

```
#qtl?
stats1 <- getm(y, x, pval=pval, qtl, model=model, p=p)

} else {
qtl <- quantile(y, c(pr, 1-pr))
y.sub <- y[y<=qtl[1]|y>=qtl[2]]
x.sub <- x[y<=qtl[1]|y>=qtl[2], ]

#Given y and x
stats1 <- getm(y.sub, x.sub, pval=pval, qtl, model=model, p=p)
}
}

if(pval)
stats[['pval']][sim,f.name[j]] <- stats1 else
stats[['pval']][sim,f.name[j]] <- stats1
#stats[['pval']][[paste(f.name[j])]][sim,f.name[j]] <- stats1 else
#stats[['pval']][[paste(f.name[j])]][sim,f.name[j]] <- stats1

##4) permutation

##Permute the lables of samples
#    index <- sample(x=rownames(x.sub), size=nrow(x.sub), replace=FALSE)
#    per.y <- y.sub[index]
```

```
    ##get the distribution of tmax under null
   #  stats2 <- NULL

    #  stats2 <- sapply("snp1", function(i){getm(y=per.y, x.sub, i, pval=pval)})

    #if(pval)
     # tmax[j,sim] <- stats2 else
      #tmax[j,sim] <- stats2
      #}
    }
   }

 #get the test statistic/pval for 2)
 #for(pr in p){
  # j <- which(pr%in%p)
   #stats[['Perm']][ ,f.name[pr]] <- sapply(stats[['Bonf']][,"snp1"], function(x){ifelse(pval,
sum(tmax[j,]<=x), sum(tmax[j,]>=x))})
 #}
 stats
}

#small functions
#function to run anova for a particular phenotype p with snp i. Output could be either p-value or F-stats
getm <- function(y, x, pval=TRUE, qtl, model=model, p){
 if(p==1){
```

```
  mod <- aov(y ~ as.factor(x))
    if(pval)
      stats <- summary(mod)[[1]]$P[1] else
      stats <- summary(mod)[[1]]$F[1]

} else {

tab <- matrix(0, nrow=2, ncol=3, dimnames=list(c("cases", "controls"), c("0", "1", "2")))
tab1 <- table(x[y>=qtl[2]])
tab2 <- table(x[y<=qtl[1]])
tab[1,names(tab1)] <- tab1
tab[2,names(tab2)] <- tab2
  if(pval)
    stats <- fisher.test(tab)$p
}
Stats }
```

```
# Runfile: Power analysis of different ways extreme case-control design.

# Written by: Chen Suo


rm(list=ls())


library(MASS) #this package allows us to use function mvrnorm and has already been installed as default in R

#To instal 'Rlab'. Run the following two lines:

#source("http://www.bioconductor.org/biocLite.R")

#biocLite("Rlab")

#library(Rlab) # generates Bernoulli random deviates


setwd("E:\\Suochen\\scientific reports\\rebuttall\\source_code")  #Please change to your own directory

source('batch_extreQTL_simu.r')


#AA, Aa, aa

wd <- getwd()

nsim <- 10000

m <- 1

pval <- TRUE

norm <- TRUE

cali <- TRUE

n <- 300

p <- seq(0.1, 0.5, length.out=5)

nf <- length(p)

f.name <- sapply(c(1:nf), function(x){paste("f", round(x, 2), sep="")})
```

```
cols <- c(1, 2, 6, 4, 7)

q1 <- 0.3


#deltas <- c(1.4, 1.3, 1.2, 1)

deltas <- seq(1, 1.4, length.out=5)

pw <-  matrix(NA, ncol=length(p), nrow=length(deltas), dimnames=list(deltas, f.name))

model <- 'multiplicative'


delta <- 1

stats.null <- simuXY(nsim=nsim, n=n, m=m, delta=delta, mu=c(1*delta^2, 1*delta, 1), p=p, q1=q1, SD=1,
pval=pval, norm=norm, seed=set.seed(2811), model=model,random_sampling=FALSE)


cutoff <- matrix(NA, ncol=1, nrow=length(p), dimnames=list(f.name, paste(delta)))


  for(pr in p){

   j <- which(p%in%pr)

   for(i in 1:1){

     if(pval){

      if(cali)

       cutoff[j, i] <- quantile(stats.null[['pval']][,j], probs=0.05) else

       cutoff[j, i] <- 0.05

     } else {

      if(cali)

       cutoff[j, i] <- quantile(stats.null[['pval']][,j], probs=0.95) else

       cutoff[j, i] <- 0.05

      }
```

```
    }
   }

stats <- NULL
for(delta in deltas){
stats[[paste(delta)]] <- simuXY(nsim=nsim, n=n, m=m, delta=delta, p=p, mu=c(1*delta^2, 1*delta, 1),
q1=q1, SD=1, pval=pval, norm=norm, seed=set.seed(2811), model=model, random_sampling=FALSE)

for(pr in p){
   j <- which(p%in%pr)
   if(pval)
      #pw[paste(delta),j] <- sum(stats[[paste(delta)]][['pval']][,j] <= 0.05)/nsim else
      pw[paste(delta),j] <- sum(stats[[paste(delta)]][['pval']][,j] <= cutoff[j, 1])/nsim else
      pw[paste(delta),j] <- sum(stats[[paste(delta)]][['pval']] >= cutoff[j,i])/nsim

}
}

#quantitative
delta <- 1
stats.null.q <- simuXY(nsim=nsim, n=n, m=m, delta=delta, mu=c(1*delta^2, 1*delta, 1), p=1, q1=q1,
SD=1, pval=pval, norm=norm, seed=set.seed(2811), model=model)
stats.q <- NULL
for(delta in deltas){
  stats.q[[paste(delta)]] <- simuXY(nsim=nsim, n=n, m=m, delta=delta, p=1, mu=c(1*delta^2, 1*delta, 1),
q1=q1, SD=1, pval=pval, norm=norm, seed=set.seed(2811), model=model, random_sampling=FALSE)
}
```

```
cutoff.q <- quantile(stats.null.q[['pval']][,1], probs=0.05)

pw.q <- sapply(c(1:length(deltas)), function(j) sum(stats.q[[j]][['pval']][,1] <= cutoff.q)/nsim)


#plot figure 1: power vs p

plot(p, pw[1,], xlab="Fractions", ylim=c(0,1), ylab="power", type="l", lty=1, col=cols[1], lwd=2,
main=paste("sample size = ", n, "; norm = ", norm, "; nsim =", nsim, "; model =", model, sep=""),
cex.main=0.8)

lines(p, pw[2,], lty=2, col=cols[2], lwd=2)

lines(p, pw[3,], lty=3, col=cols[3], lwd=2)

lines(p, pw[4,], lty=4, col=cols[4], lwd=2)

legend("bottomright", sapply(deltas, function(x) paste(expression(delta), x)), lty=c(1:4), col=cols, lwd=2)

abline(h=0.05, col='gray')


#power vs deltas

#frac <- round(p,1)

frac <- round(c(p[1], p[2], p[3], p[4], p[5]),1)

plot(deltas[length(deltas):1], pw[nrow(pw):1,1], xlab=expression(delta), ylim=c(0,1), ylab="power",
type="l", lty=1, col=cols[1], lwd=2, main=paste("sample size = ", n, "; norm = ", norm, "; nsim =", nsim, ";
model =", model, "; MAF =", q1, sep=""), cex.main=0.8)

lines(deltas[length(deltas):1], pw[nrow(pw):1,2], lty=2, col=cols[2], lwd=2)

lines(deltas[length(deltas):1], pw[nrow(pw):1,3], lty=3, col=cols[3], lwd=2)

lines(deltas[length(deltas):1], pw[nrow(pw):1,4], lty=4, col=cols[4], lwd=2)

lines(deltas[length(deltas):1], pw[nrow(pw):1,5], lty=5, col=cols[5], lwd=2)

legend("bottomright", sapply(frac, function(x) paste("fractions", x)), lty=c(1:5), col=cols, lwd=2)

points(deltas, pw.q, pch=4, lwd=2)


#Aa+aa
```

```
wd <- getwd()

nsim <- 10000

m <- 1

pval <- TRUE

norm <- TRUE

cali <- TRUE

n <- 300

p <- seq(0.1, 0.5, length.out=5)

nf <- length(p)

f.name <- sapply(c(1:nf), function(x){paste("f", round(x, 2), sep="")})

cols <- c(1, 2, 6, 4, 7)

q1 <- 0.3


#deltas <- c(1.4, 1.3, 1.2, 1)

deltas <- seq(1, 1.4, length.out=5)

pw <-  matrix(NA, ncol=length(p), nrow=length(deltas), dimnames=list(deltas, f.name))

model <- 'dominant'


delta <- 1

stats.null <- simuXY(nsim=nsim, n=n, m=m, p=p,delta=delta, mu=c(1*delta^2, 1*delta, 1), q1=q1, SD=1,
pval=pval, norm=norm, seed=set.seed(2811), model=model, random_sampling=FALSE)


cutoff <- matrix(NA, ncol=1, nrow=length(p), dimnames=list(f.name, paste(delta)))


   for(pr in p){

     j <- which(p%in%pr)
```

```
    for(i in 1:1){
      if(pval){
        if(cali)
          cutoff[j, i] <- quantile(stats.null[['pval']][,j], probs=0.05) else
          cutoff[j, i] <- 0.05
      } else {
        if(cali)
          cutoff[j, i] <- quantile(stats.null[['pval']][,j], probs=0.95) else
          cutoff[j, i] <- 0.05
        }
    }
  }


stats <- NULL
for(delta in deltas){
stats[[paste(delta)]] <- simuXY(nsim=nsim, n=n, m=m, p=p,delta=delta, mu=c(1*delta^2, 1*delta, 1),
q1=q1, SD=1, pval=pval, norm=norm, seed=set.seed(2811), model=model, random_sampling=FALSE)


for(pr in p){
  j <- which(p%in%pr)
  if(pval)
    #pw[paste(delta),j] <- sum(stats[[paste(delta)]][['pval']][,j] <= 0.05)/nsim else
    pw[paste(delta),j] <- sum(stats[[paste(delta)]][['pval']][,j] <= cutoff[j, 1])/nsim else
    pw[paste(delta),j] <- sum(stats[[paste(delta)]][['pval']] >= cutoff[j,i])/nsim


}
```

```
}

#quantitative
delta <- 1
stats.null.q <- simuXY(nsim=nsim, n=n, m=m, delta=delta, mu=c(1*delta^2, 1*delta, 1), p=1, q1=q1,
SD=1, pval=pval, norm=norm, seed=set.seed(2811), model=model, random_sampling=FALSE)
stats.q <- NULL
for(delta in deltas){
  stats.q[[paste(delta)]] <- simuXY(nsim=nsim, n=n, m=m, delta=delta, p=1, mu=c(1*delta^2, 1*delta, 1),
q1=q1, SD=1, pval=pval, norm=norm, seed=set.seed(2811), model=model, random_sampling=FALSE)
}
cutoff.q <- quantile(stats.null.q[['pval']][,1], probs=0.05)
pw.q <- sapply(c(1:length(deltas)), function(j) sum(stats.q[[j]][['pval']][,1] <= cutoff.q)/nsim)

#plot figure 1: power vs p
plot(p, pw[1,], xlab="Fractions", ylim=c(0,1), ylab="power", type="l", lty=1, col=cols[1], lwd=2,
main=paste("sample size = ", n, "; norm = ", norm, "; nsim =", nsim, "; model =", model, sep=""),
cex.main=0.8)
lines(p, pw[2,], lty=2, col=cols[2], lwd=2)
lines(p, pw[3,], lty=3, col=cols[3], lwd=2)
lines(p, pw[4,], lty=4, col=cols[4], lwd=2)
legend("bottomright", sapply(deltas, function(x) paste(expression(delta), x)), lty=c(1:4), col=cols, lwd=2)
abline(h=0.05, col='gray')

#power vs deltas
frac <- round(p,1)
#frac <- round(c(p[1], p[3], p[5], p[8], p[10]),1)
```

```
plot(deltas[length(deltas):1], pw[nrow(pw):1,1], xlab=expression(delta), ylim=c(0,1), ylab="power", type="l", lty=1, col=cols[1], lwd=2, main=paste("sample size = ", n, "; norm = ", norm, "; nsim =", nsim, "; model =", model, "; MAF =", q1, sep=""), cex.main=0.8)

lines(deltas[length(deltas):1], pw[nrow(pw):1,2], lty=2, col=cols[2], lwd=2)

lines(deltas[length(deltas):1], pw[nrow(pw):1,3], lty=3, col=cols[3], lwd=2)

lines(deltas[length(deltas):1], pw[nrow(pw):1,4], lty=4, col=cols[4], lwd=2)

lines(deltas[length(deltas):1], pw[nrow(pw):1,5], lty=5, col=cols[5], lwd=2)

legend("bottomright", sapply(frac, function(x) paste("fractions", x)), lty=c(1:5), col=cols, lwd=2)

points(deltas, pw.q, pch=4, lwd=2)


#aa. a is the minor allele

wd <- getwd()

nsim <- 10000

m <- 1

pval <- TRUE

norm <- TRUE

cali <- TRUE

n <- 300

p <- seq(0.1, 0.5, length.out=5)

nf <- length(p)

f.name <- sapply(c(1:nf), function(x){paste("f", round(x, 2), sep="")})

cols <- c(1, 2, 6, 4, 7)

q1 <- 0.3


#deltas <- c(1.7, 1.5, 1.3, 1)

deltas <- seq(1, 1.7, length.out=5)
```

```
pw <-  matrix(NA, ncol=length(p), nrow=length(deltas), dimnames=list(deltas, f.name))

model <- 'recessive'


delta <- 1

stats.null <- simuXY(nsim=nsim, n=n, m=m, delta=delta, mu=c(1*delta^2, 1*delta, 1), p=p, q1=q1, SD=1,
pval=pval, norm=norm, seed=set.seed(2811), model=model, random_sampling=FALSE)


cutoff <- matrix(NA, ncol=1, nrow=length(p), dimnames=list(f.name, paste(delta)))


  for(pr in p){

    j <- which(p%in%pr)

    for(i in 1:1){

        if(pval){

          if(cali)

            cutoff[j, i] <- quantile(stats.null[['pval']][,j], probs=0.05) else

            cutoff[j, i] <- 0.05

        } else {

          if(cali)

            cutoff[j, i] <- quantile(stats.null[['pval']][,j], probs=0.95) else

            cutoff[j, i] <- 0.05

          }

    }

  }


stats <- NULL

for(delta in deltas){
```

```
stats[[paste(delta)]] <- simuXY(nsim=nsim, p=p,n=n, m=m, delta=delta, mu=c(1*delta^2, 1*delta, 1),
q1=q1, SD=1, pval=pval, norm=norm, seed=set.seed(2811), model=model, random_sampling=FALSE)

for(pr in p){
  j <- which(p%in%pr)
  if(pval)
    #pw[paste(delta),j] <- sum(stats[[paste(delta)]][['pval']][,j] <= 0.05)/nsim else
    pw[paste(delta),j] <- sum(stats[[paste(delta)]][['pval']][,j] <= cutoff[j, 1])/nsim else
    pw[paste(delta),j] <- sum(stats[[paste(delta)]][['pval']] >= cutoff[j,i])/nsim

}
}

#quantitative
delta <- 1
stats.null.q <- simuXY(nsim=nsim, n=n, m=m, delta=delta, mu=c(1*delta^2, 1*delta, 1), p=1, q1=q1,
SD=1, pval=pval, norm=norm, seed=set.seed(2811), model=model, random_sampling=FALSE)
stats.q <- NULL
for(delta in deltas){
 stats.q[[paste(delta)]] <- simuXY(nsim=nsim, n=n, m=m, delta=delta, p=1, mu=c(1*delta^2, 1*delta, 1),
q1=q1, SD=1, pval=pval, norm=norm, seed=set.seed(2811), model=model, random_sampling=FALSE)
}
cutoff.q <- quantile(stats.null.q[['pval']][,1], probs=0.05)
pw.q <- sapply(c(1:length(deltas)), function(j) sum(stats.q[[j]][['pval']][,1] <= cutoff.q)/nsim)

#plot figure 1: power vs p
```

```
plot(p, pw[1,], xlab="Fractions", ylim=c(0,1), ylab="power", type="l", lty=1, col=cols[1], lwd=2,
main=paste("sample size = ", n, "; norm = ", norm, "; nsim =", nsim, "; model =", model, sep=""),
cex.main=0.8)

lines(p, pw[2,], lty=2, col=cols[2], lwd=2)

lines(p, pw[3,], lty=3, col=cols[3], lwd=2)

lines(p, pw[4,], lty=4, col=cols[4], lwd=2)

legend("bottomright", sapply(deltas, function(x) paste(expression(delta), x)), lty=c(1:4), col=cols, lwd=2)

abline(h=0.05, col='gray')


#power vs deltas

frac <- round(p,1)

#frac <- round(c(p[1], p[3], p[5], p[8], p[10]),1)

plot(deltas[length(deltas):1], pw[nrow(pw):1,1], xlab=expression(delta), ylim=c(0,1), ylab="power",
type="l", lty=1, col=cols[1], lwd=2, main=paste("sample size = ", n, "; norm = ", norm, "; nsim =", nsim, ";
model =", model, "; MAF =", q1, sep=""), cex.main=0.8)

lines(deltas[length(deltas):1], pw[nrow(pw):1,2], lty=2, col=cols[2], lwd=2)

lines(deltas[length(deltas):1], pw[nrow(pw):1,3], lty=3, col=cols[3], lwd=2)

lines(deltas[length(deltas):1], pw[nrow(pw):1,4], lty=4, col=cols[4], lwd=2)

lines(deltas[length(deltas):1], pw[nrow(pw):1,5], lty=5, col=cols[5], lwd=2)

legend("bottomright", sapply(frac, function(x) paste("fractions", x)), lty=c(1:5), col=cols, lwd=2)

points(deltas, pw.q, pch=4, lwd=2)




source('batch_extreQTL_simu.r')

#decrease MAF s.t pheno distribution becomes skewed

#AA, Aa, aa

wd <- getwd()
```

```
nsim <- 10000

m <- 1

pval <- TRUE

norm <- TRUE

cali <- TRUE

n <- 500

p <- seq(0.1, 0.5, length.out=5)

q1 <- 0.2

nf <- length(p)

f.name <- sapply(c(1:nf), function(x){paste("f", round(x, 2), sep="")})

cols <- c(1, 2, 6, 4, 7)


#deltas <- c(1.4, 1.3, 1.2, 1)

deltas <- seq(1, 1.4, length.out=5)

pw <-  matrix(NA, ncol=length(p), nrow=length(deltas), dimnames=list(deltas, f.name))

model <- 'multiplicative'


delta <- 1

stats.null <- simuXY(nsim=nsim, n=n, m=m, delta=delta, mu=c(1*delta^2, 1*delta, 1), p=p, q1=q1, SD=1,
pval=pval, norm=norm, seed=set.seed(2811), model=model, random_sampling=FALSE)


cutoff <- matrix(NA, ncol=1, nrow=length(p), dimnames=list(f.name, paste(delta)))


  for(pr in p){

    j <- which(p%in%pr)

    for(i in 1:1){
```

```
        if(pval){
          if(cali)
            cutoff[j, i] <- quantile(stats.null[['pval']][,j], probs=0.05) else
            cutoff[j, i] <- 0.05
        } else {
          if(cali)
            cutoff[j, i] <- quantile(stats.null[['pval']][,j], probs=0.95) else
            cutoff[j, i] <- 0.05
          }
      }
    }

stats <- NULL
for(delta in deltas){
stats[[paste(delta)]] <- simuXY(nsim=nsim, n=n, m=m, delta=delta,p=p, mu=c(1*delta^2, 1*delta, 1),
q1=q1, SD=1, pval=pval, norm=norm, seed=set.seed(2811), model=model, random_sampling=FALSE)

for(pr in p){
   j <- which(p%in%pr)
   if(pval)
       #pw[paste(delta),j] <- sum(stats[[paste(delta)]][['pval']][,j] <= 0.05)/nsim else
       pw[paste(delta),j] <- sum(stats[[paste(delta)]][['pval']][,j] <= cutoff[j, 1])/nsim else
       pw[paste(delta),j] <- sum(stats[[paste(delta)]][['pval']] >= cutoff[j,i])/nsim

}
}
```

```
#quantitative

delta <- 1

stats.null.q <- simuXY(nsim=nsim, n=n, m=m, delta=delta, mu=c(1*delta^2, 1*delta, 1), p=1, q1=q1,
SD=1, pval=pval, norm=norm, seed=set.seed(2811), model=model, random_sampling=FALSE)

stats.q <- NULL

for(delta in deltas){

  stats.q[[paste(delta)]] <- simuXY(nsim=nsim, n=n, m=m, delta=delta, p=1, mu=c(1*delta^2, 1*delta, 1),
q1=q1, SD=1, pval=pval, norm=norm, seed=set.seed(2811), model=model, random_sampling=FALSE)

}

cutoff.q <- quantile(stats.null.q[['pval']][,1], probs=0.05)

pw.q <- sapply(c(1:length(deltas)), function(j) sum(stats.q[[j]][['pval']][,1] <= cutoff.q)/nsim)


#plot figure 1: power vs r

plot(p, pw[1,], xlab="Fractions", ylim=c(0,1), ylab="power", type="l", lty=1, col=cols[1], lwd=2,
main=paste("sample size = ", n, "; ", "; norm = ", norm, "; nsim =", nsim, "; model =", model, sep=""),
cex.main=0.8)

lines(p, pw[2,], lty=2, col=cols[2], lwd=2)

lines(p, pw[3,], lty=3, col=cols[3], lwd=2)

lines(p, pw[4,], lty=4, col=cols[4], lwd=2)

legend("bottomright", sapply(deltas, function(x) paste(expression(delta), x)), lty=c(1:4), col=cols, lwd=2)

abline(h=0.05, col='gray')


#power vs deltas

frac <- round(p,1)

#frac <- round(c(p[1], p[3], p[5], p[8], p[10]),1)
```

```
plot(deltas[length(deltas):1], pw[nrow(pw):1,1], xlab=expression(delta), ylim=c(0,1), ylab="power", type="l", lty=1, col=cols[1], lwd=2, main=paste("sample size = ", n, "; norm = ", norm, "; nsim =", nsim, "; model =", model, "; MAF =", q1, sep=""), cex.main=0.8)

lines(deltas[length(deltas):1], pw[nrow(pw):1,2], lty=2, col=cols[2], lwd=2)

lines(deltas[length(deltas):1], pw[nrow(pw):1,3], lty=3, col=cols[3], lwd=2)

lines(deltas[length(deltas):1], pw[nrow(pw):1,4], lty=4, col=cols[4], lwd=2)

lines(deltas[length(deltas):1], pw[nrow(pw):1,5], lty=5, col=cols[5], lwd=2)

legend("bottomright", sapply(frac, function(x) paste("fractions", x)), lty=c(1:5), col=cols, lwd=2)

points(deltas, pw.q, pch=4, lwd=2)


#Aa+aa. a is the minor allele

source('batch_extreQTL_simu.r')

wd <- getwd()

nsim <- 10000

m <- 1

pval <- TRUE

norm <- TRUE

cali <- TRUE

n <- 500

p <- seq(0.1, 0.5, length.out=5)

nf <- length(p)

f.name <- sapply(c(1:nf), function(x){paste("f", round(x, 2), sep="")})

cols <- c(1, 2, 6, 4, 7)

q1 <- 0.2


#deltas <- c(1.4, 1.3, 1.2, 1)
```

```
deltas <- seq(1, 1.4, length.out=5)

pw <-  matrix(NA, ncol=length(p), nrow=length(deltas), dimnames=list(deltas, f.name))

model <- 'dominant'


delta <- 1

stats.null <- simuXY(nsim=nsim, n=n, m=m, p=p,delta=delta, mu=c(1*delta^2, 1*delta, 1), q1=q1, SD=1,
pval=pval, norm=norm, seed=set.seed(2811), model=model, random_sampling=FALSE)


cutoff <- matrix(NA, ncol=1, nrow=length(p), dimnames=list(f.name, paste(delta)))


  for(pr in p){

   j <- which(p%in%pr)

   for(i in 1:1){

      if(pval){

       if(cali)

         cutoff[j, i] <- quantile(stats.null[['pval']][,j], probs=0.05) else

         cutoff[j, i] <- 0.05

      } else {

       if(cali)

         cutoff[j, i] <- quantile(stats.null[['pval']][,j], probs=0.95) else

         cutoff[j, i] <- 0.05

       }

   }

  }


stats <- NULL
```

```
for(delta in deltas){

stats[[paste(delta)]] <- simuXY(nsim=nsim, n=n, m=m, p=p,delta=delta, mu=c(1*delta^2, 1*delta, 1),
q1=q1, SD=1, pval=pval, norm=norm, seed=set.seed(2811), model=model, random_sampling=FALSE)


for(pr in p){

  j <- which(p%in%pr)

  if(pval)

    #pw[paste(delta),j] <- sum(stats[[paste(delta)]][['pval']][,j] <= 0.05)/nsim else

    pw[paste(delta),j] <- sum(stats[[paste(delta)]][['pval']][,j] <= cutoff[j, 1])/nsim else

    pw[paste(delta),j] <- sum(stats[[paste(delta)]][['pval']] >= cutoff[j,i])/nsim


}

}


#quantitative

delta <- 1

stats.null.q <- simuXY(nsim=nsim, n=n, m=m, delta=delta, mu=c(1*delta^2, 1*delta, 1), p=1, q1=q1,
SD=1, pval=pval, norm=norm, seed=set.seed(2811), model=model, random_sampling=FALSE)

stats.q <- NULL


for(delta in deltas){

 stats.q[[paste(delta)]] <- simuXY(nsim=nsim, n=n, m=m, delta=delta, p=1, mu=c(1*delta^2, 1*delta, 1),
q1=q1, SD=1, pval=pval, norm=norm, seed=set.seed(2811), model=model, random_sampling=FALSE)

}

cutoff.q <- quantile(stats.null.q[['pval']][,1], probs=0.05)

pw.q <- sapply(c(1:length(deltas)), function(j) sum(stats.q[[j]][['pval']][,1] <= cutoff.q)/nsim)
```

```
#plot figure 1: power vs p

plot(p, pw[1,], xlab="Fractions", ylim=c(0,1), ylab="power", type="l", lty=1, col=cols[1], lwd=2,
main=paste("sample size = ", n, "; norm = ", norm, "; nsim =", nsim, "; model =", model, sep=""),
cex.main=0.8)

lines(p, pw[2,], lty=2, col=cols[2], lwd=2)

lines(p, pw[3,], lty=3, col=cols[3], lwd=2)

lines(p, pw[4,], lty=4, col=cols[4], lwd=2)

legend("bottomright", sapply(deltas, function(x) paste(expression(delta), x)), lty=c(1:4), col=cols, lwd=2)

abline(h=0.05, col='gray')


#power vs deltas

#frac <- round(p,1)

frac <- round(c(p[1], p[3], p[5], p[8], p[10]),1)

plot(deltas[length(deltas):1], pw[nrow(pw):1,1], xlab=expression(delta), ylim=c(0,1), ylab="power",
type="l", lty=1, col=cols[1], lwd=2, main=paste("sample size = ", n, "; norm = ", norm, "; nsim =", nsim, ";
model =", model, "; MAF =", q1, sep=""), cex.main=0.8)

lines(deltas[length(deltas):1], pw[nrow(pw):1,3], lty=2, col=cols[2], lwd=2)

lines(deltas[length(deltas):1], pw[nrow(pw):1,5], lty=3, col=cols[3], lwd=2)

lines(deltas[length(deltas):1], pw[nrow(pw):1,8], lty=4, col=cols[4], lwd=2)

lines(deltas[length(deltas):1], pw[nrow(pw):1,10], lty=5, col=cols[5], lwd=2)

legend("bottomright", sapply(frac, function(x) paste("fractions", x)), lty=c(1:5), col=cols, lwd=2)

points(deltas, pw.q, pch=4, lwd=2)


#aa. a is the minor allele

wd <- getwd()

source('batch_extreQTL_simu.r')

nsim <- 10000
```

```
m <- 1

pval <- TRUE

norm <- TRUE

cali <- TRUE

n <- 500

p <- seq(0.1, 0.5, length.out=5)

nf <- length(p)

f.name <- sapply(c(1:nf), function(x){paste("f", round(x, 2), sep="")})

cols <- c(1, 2, 6, 4, 7)

q1 <- 0.2


deltas <- seq(1, 1.7, length.out=5)

#deltas <- c(1.7, 1.5, 1.3, 1)

pw <-  matrix(NA, ncol=length(p), nrow=length(deltas), dimnames=list(deltas, f.name))

model <- 'recessive'


delta <- 1

stats.null <- simuXY(nsim=nsim, n=n, m=m, delta=delta, mu=c(1*delta^2, 1*delta, 1), p=p, q1=q1, SD=1,
pval=pval, norm=norm, seed=set.seed(2811), model=model, random_sampling=FALSE)


cutoff <- matrix(NA, ncol=1, nrow=length(p), dimnames=list(f.name, paste(delta)))


  for(pr in p){

   j <- which(p%in%pr)

   for(i in 1:1){

     if(pval){
```

```
      if(cali)
        cutoff[j, i] <- quantile(stats.null[['pval']][,j], probs=0.05) else
        cutoff[j, i] <- 0.05
     } else {
      if(cali)
        cutoff[j, i] <- quantile(stats.null[['pval']][,j], probs=0.95) else
        cutoff[j, i] <- 0.05
      }
   }
  }

stats <- NULL
for(delta in deltas){
stats[[paste(delta)]] <- simuXY(nsim=nsim, p=p,n=n, m=m, delta=delta, mu=c(1*delta^2, 1*delta, 1),
q1=q1, SD=1, pval=pval, norm=norm, seed=set.seed(2811), model=model, random_sampling=FALSE)

for(pr in p){
  j <- which(p%in%pr)
  if(pval)
    #pw[paste(delta),j] <- sum(stats[[paste(delta)]][['pval']][,j] <= 0.05)/nsim else
    pw[paste(delta),j] <- sum(stats[[paste(delta)]][['pval']][,j] <= cutoff[j, 1])/nsim else
    pw[paste(delta),j] <- sum(stats[[paste(delta)]][['pval']] >= cutoff[j,i])/nsim

}
}
```

```
#quantitative

delta <- 1

stats.null.q <- simuXY(nsim=nsim, n=n, m=m, delta=delta, mu=c(1*delta^2, 1*delta, 1), p=1, q1=q1, SD=1, pval=pval, norm=norm, seed=set.seed(2811), model=model, random_sampling=FALSE)

stats.q <- NULL

for(delta in deltas){

  stats.q[[paste(delta)]] <- simuXY(nsim=nsim, n=n, m=m, delta=delta, p=1, mu=c(1*delta^2, 1*delta, 1), q1=q1, SD=1, pval=pval, norm=norm, seed=set.seed(2811), model=model, random_sampling=FALSE)

}

cutoff.q <- quantile(stats.null.q[['pval']][,1], probs=0.05)

pw.q <- sapply(c(1:length(deltas)), function(j) sum(stats.q[[j]][['pval']][,1] <= cutoff.q)/nsim)


#plot figure 1: power vs p

plot(p, pw[1,], xlab="Fractions", ylim=c(0,1), ylab="power", type="l", lty=1, col=cols[1], lwd=2, main=paste("sample size = ", n, "; norm = ", norm, "; nsim =", nsim, "; model =", model, sep=""), cex.main=0.8)

lines(p, pw[2,], lty=2, col=cols[2], lwd=2)

lines(p, pw[3,], lty=3, col=cols[3], lwd=2)

lines(p, pw[4,], lty=4, col=cols[4], lwd=2)

legend("bottomright", sapply(deltas, function(x) paste(expression(delta), x)), lty=c(1:4), col=cols, lwd=2)

abline(h=0.05, col='gray')


#power vs deltas

frac <- round(p,1)

#frac <- round(c(p[1], p[3], p[5], p[8], p[10]),1)

plot(deltas[length(deltas):1], pw[nrow(pw):1,1], xlab=expression(delta), ylim=c(0,1), ylab="power", type="l", lty=1, col=cols[1], lwd=2, main=paste("sample size = ", n, "; norm = ", norm, "; nsim =", nsim, "; model =", model, "; MAF =", q1, sep=""), cex.main=0.8)
```

```
lines(deltas[length(deltas):1], pw[nrow(pw):1,2], lty=2, col=cols[2], lwd=2)

lines(deltas[length(deltas):1], pw[nrow(pw):1,3], lty=3, col=cols[3], lwd=2)

lines(deltas[length(deltas):1], pw[nrow(pw):1,4], lty=4, col=cols[4], lwd=2)

lines(deltas[length(deltas):1], pw[nrow(pw):1,5], lty=5, col=cols[5], lwd=2)

legend("bottomright", sapply(frac, function(x) paste("fractions", x)), lty=c(1:5), col=cols, lwd=2)

points(deltas, pw.q, pch=4, lwd=2)
```
